# Supplementary material for: The causal relationship between the human gut microbiota and pyogenic arthritis: a Mendelian randomization study
Source: Front Cell Infect Microbiol. 2024 Nov 26;14:1452480. doi: 10.3389/fcimb.2024.1452480 (PMC11629706; doi:10.3389/fcimb.2024.1452480)
Supplement: Supplementary file 1 [file DataSheet1.zip › mendelian test/ebi-a-GCST90027697.csv_forest.pdf]

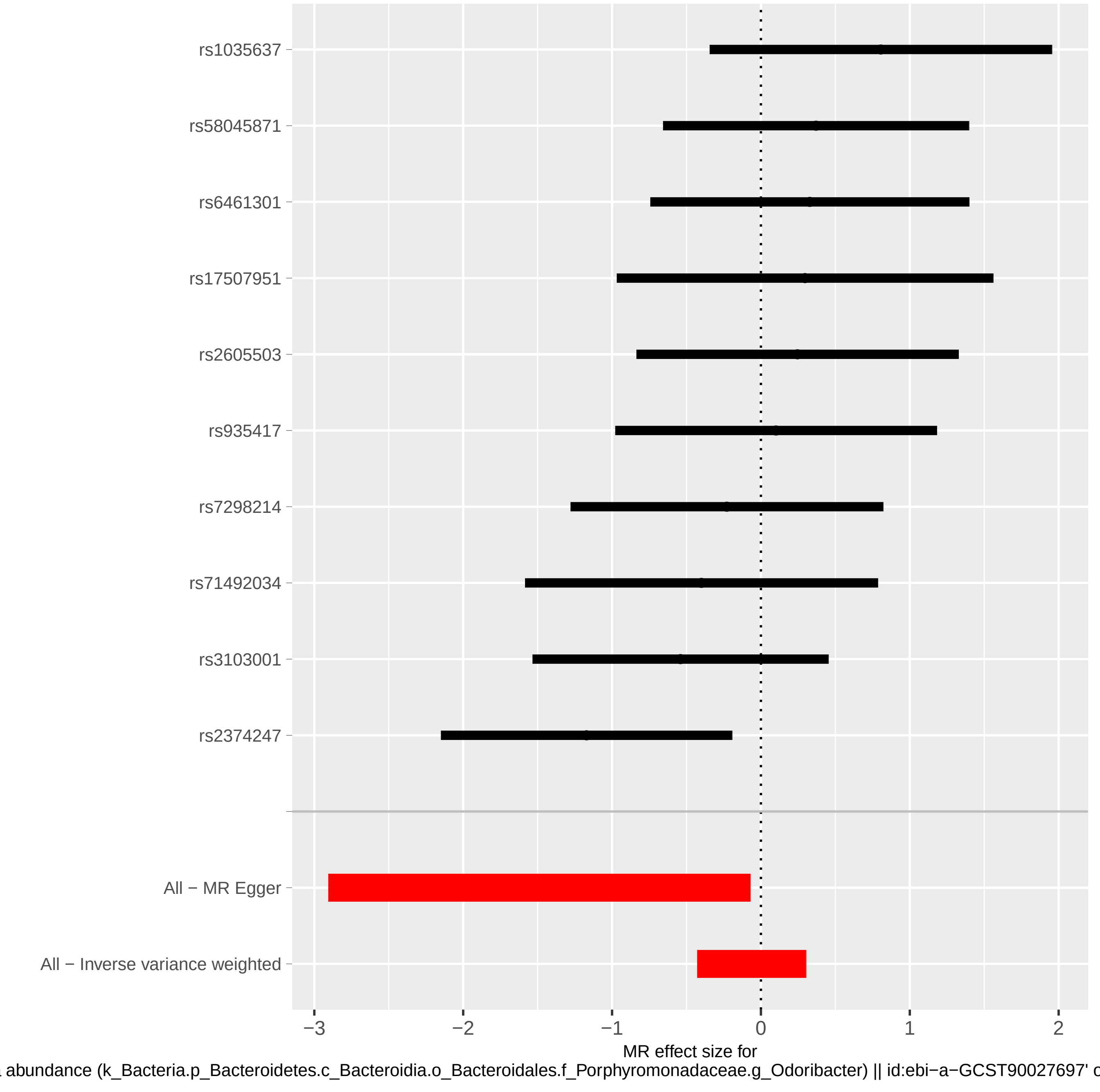

rs1035637
rs58045871
rs6461301
rs17507951
rs2605503
rs935417
rs7298214
rs71492034
rs3103001
rs2374247
All − MR Egger
All − Inverse variance weighted
−3
−2
−1
0
1
2
MR effect size for
abundance (k_Bacteria.p_Bacteroidetes.c_Bacteroidia.o_Bacteroidales.f_Porphyromonadaceae.g_Odoribacter) || id:ebi−a−GCST90027697' o
